# Heterogenous Immune Response to TB is Not a False Negative

**DOI:** 10.4269/ajtmh.19-0896a

**Published:** 2020-03

**Authors:** Andrew DiNardo

**Affiliations:** Department of Pediatrics; The Global Tuberculosis Program; Texas Children’s Hospital; Immigrant and Global Health; Baylor College of Medicine; Houston, Texas; E-mail: dinardo@bcm.edu

Dear Sir,

The article by Drs. Wang and Liu^1^ should be commended for evaluating the epidemiologic risks for culture-positive pediatric tuberculosis (TB) with a negative interferon-gamma release assay (IGRA). Pediatric TB is notoriously challenging to diagnose, with the minority of cases microbiologically confirmed by culture or Xpert.^2^ Therefore, identifying younger age, lower weight, and hypoproteinemia as risk factors for a negative IGRA is beneficial to clinicians.

However, I would warn against calling the IGRA “false negative” as this suggests a fault of the assay and not a clinically relevant aberration in the immune system. In fact, the assay is likely operating as it should, identifying IFN-γ production from immune cells after stimulation with *Mtb*-specific peptides (ESAT-6 and CFP-10) and mitogen stimulation. Since the 1940s, we have known that 5–25% of TB patients fail to mount a cell-mediated immune response to *Mtb* antigens or nonspecific stimuli, such as Candida and histoplasmin antigens.^3–10^ TB induces immune suppression and anergy, and in this setting, a negative test should not be referred to as a “false-negative” assay. By contrast, this is clinically interesting: why are some TB patients anergic and more importantly, how should they be treated? Despite most TB patients becoming culture negative 4–8 weeks after commencing anti-TB therapy (ATT), therapy must be continued for 6 months so that subculturable amounts of *Mtb* do not cause disease relapse.^11,12^ With evidence that > 85% of TB patients could successfully be treated with only 4 months of ATT,^13^ we should evaluate how IGRA status determines the requisite duration of ATT. Similarly, with increasing work to identify host-directed therapies to improve ATT, it is likely that IGRA-negative TB patients will need to be considered differently than TB patients able to mount antigen-specific IFN-γ production. TB is a heterogenous disease, with the ability of host immunity to produce IFN-γ likely critical in stratifying clinical outcomes.

Compared with IGRA-positive individuals with TB, IGRA-negative TB patients have worse outcomes^14^ and, therefore, deserve increased clinical attention, and future researchers should address the biologic underpinnings of their immune suppression. Instead of labeling it “false negative,” we should embrace characterizing the complexity of the disease process and continue research as implemented by Wang and Liu,^1^ while also exploring the clinical implications. If the authors have access to clinical outcome data, was IGRA-status associated with slower time to culture conversion or increased risk of treatment failure?

## References

[1] Wang MS Liu XJ, 2019 Risk factors for false-negative interferon-gamma release assay results in culture-confirmed childhood TB. Am J Trop Med Hyg 101: 1303–1307. 31674295 10.4269/ajtmh.18-0684 PMC6896862

[2] Detjen AK DiNardo AR Leyden J Steingart KR Menzies D Schiller I Dendukuri N Mandalakas AM, 2015 Xpert MTB/RIF assay for the diagnosis of pulmonary tuberculosis in children: a systematic review and meta-analysis. Lancet Respir Med 3: 451–461. 25812968 10.1016/S2213-2600(15)00095-8 PMC4756280

[3] Zeitz SJ Ostrow JH Van Arsdel PP Jr., 1974 Humoral and cellular immunity in the anergic tuberculosis patient. A prospective study. J Allergy Clin Immunol 53: 20–26. 4202828 10.1016/0091-6749(74)90095-5

[4] Holden M Dubin MR Diamond PH, 1971 Frequency of negative intermediate-strength tuberculin sensitivity in patients with active tuberculosis. N Engl J Med 285: 1506–1509. 5127141 10.1056/NEJM197112302852704

[5] Michael L Furcolow BH Nelson WE Palmer CE, 1941 Quantitative studies of the tuberculin reaction: i. Titration of tuberculin sensitivity and its relation to tuberculous infection. Public Health Rep 56: 1082–1100.

[6] Cobelens FG Egwaga SM van Ginkel T Muwinge H Matee MI Borgdorff MW, 2006 Tuberculin skin testing in patients with HIV infection: limited benefit of reduced cutoff values. Clin Infect Dis 43: 634–639. 16886159 10.1086/506432

[7] Nash DR Douglass JE, 1980 Anergy in active pulmonary tuberculosis. A comparison between positive and negative reactors and an evaluation of 5 TU and 250 TU skin test doses. Chest 77: 32–37. 7351142 10.1378/chest.77.1.32

[8] Sollai S Galli L de Martino M Chiappini E, 2014 Systematic review and meta-analysis on the utility of interferon-gamma release assays for the diagnosis of *Mycobacterium tuberculosis* infection in children: a 2013 update. BMC Infect Dis 14 (Suppl 1): S6. 10.1186/1471-2334-14-S1-S6 PMC4016555 24564486

[9] Metcalfe JZ Everett CK Steingart KR Cattamanchi A Huang L Hopewell PC Pai M, 2011 Interferon-gamma release assays for active pulmonary tuberculosis diagnosis in adults in low- and middle-income countries: systematic review and meta-analysis. J Infect Dis 204 (Suppl 4): S1120–S1129. 21996694 10.1093/infdis/jir410 PMC3192542

[10] McMurray DN Echeverri A, 1978 Cell-mediated immunity in anergic patients with pulmonary tuberculosis. Am Rev Respir Dis 118: 827–834. 736354 10.1164/arrd.1978.118.5.827

[11] Merle CS 2014 A four-month gatifloxacin-containing regimen for treating tuberculosis. N Engl J Med 371: 1588–1598. 25337748 10.1056/NEJMoa1315817

[12] Gillespie SH Crook AM McHugh TD Mendel CM Meredith SK Murray SR Pappas F Phillips PP Nunn AJ Consortium RE, 2014 Four-month moxifloxacin-based regimens for drug-sensitive tuberculosis. N Engl J Med 371: 1577–1587. 25196020 10.1056/NEJMoa1407426 PMC4277680

[13] Imperial MZ 2018 A patient-level pooled analysis of treatment-shortening regimens for drug-susceptible pulmonary tuberculosis. Nat Med 24: 1708–1715. 30397355 10.1038/s41591-018-0224-2 PMC6685538

[14] Nguyen DT Teeter LD Graves J Graviss EA, 2018 Characteristics associated with negative interferon-gamma release assay results in culture-confirmed tuberculosis patients, Texas, USA, 2013–2015. Emerg Infect Dis 24: 534–540. 29460756 10.3201/eid2403.171633 PMC5823348

